# Powerflow Doppler Ultrasonography in the Evaluation of Mares with and Without Endometritis

**DOI:** 10.3390/vetsci12100941

**Published:** 2025-09-28

**Authors:** Camila Silva Costa Ferreira, Aline Emerim Pinna, Isadora Pires Ferreira dos Santos, Maria Clara Rangel Dias, Natália Sales Leal dos Santos, Samanta da Silva Bragueroli, Petruska Montezuma Quintino, Giovanna Brito Almeida, Bruno de Araújo Penna, Elisabeth Martins da Silva da Rocha, Guilherme Nunes de Souza, Celso Guimarães Barbosa, Vera Lucia Teixeira de Jesus, Julio César Ferraz Jacob

**Affiliations:** 1 Department of Animal Reproduction of the Institute of Animal Science, Federal Rural University of Rio de Janeiro, Seropédica 23897-000, RJ, Brazil; isadorapires.vet@gmail.com (I.P.F.d.S.); mclararangel@outlook.com.br (M.C.R.D.); natysales77@hotmail.com (N.S.L.d.S.); samantabragueroli@gmail.com (S.d.S.B.); petruska_montezuma@yahoo.com.br (P.M.Q.); celsogb1@hotmail.com (C.G.B.); jesus@ufrrj.br (V.L.T.d.J.); juliorep@ufrrj.br (J.C.F.J.); 2 Veterinary Clinic and Pathology, Fluminense Federal University, Niterói 24220-900, RJ, Brazil; aepinna@id.uff.br (A.E.P.); giovannaba2000@gmail.com (G.B.A.); bpenna@id.uff.br (B.d.A.P.); martinselisabeth@id.uff.br (E.M.d.S.d.R.); guilherme.nunes@ufjf.br (G.N.d.S.)

**Keywords:** equine, uterine pathology, reproduction

## Abstract

**Simple Summary:**

Equine endometritis is considered the main reproductive pathology in the species and the cause of the greatest economic losses in reproduction due to early pregnancy failure. However, there are no reports on the use of Powerflow Doppler ultrasound (US) in the diagnosis of endometritis when evaluating mares with or without endometritis. Therefore, our objective was to evaluate uterine blood flow using Powerflow Doppler ultrasound, correlating it with the estrous cycle days of mares with endometritis. The mares were divided into three groups: CG—control group with mares without endometritis; GES—group of mares with subclinical endometritis; and GEC—group of mares with clinical endometritis. After ovulation induction, the mares underwent transrectal B-mode US and uterine Powerflow Doppler ultrasound. Examinations were performed daily until the next ovulation. Based on these examinations, it can be observed that the subjective assessment, performed by Powerflow Doppler ultrasound, proved effective in detecting mares with clinical endometritis examined on days 1, 9, and 10 of the estrous cycle. It was found that the subjective and objective assessment of pixel quantity and intensity are interconnected in the evaluations. Therefore, it is concluded that Powerflow Doppler ultrasound can be used to detect mares with clinical endometritis.

**Abstract:**

There are no reports on the application of Powerflow mode Doppler ultrasound (US) in the diagnosis of endometritis when evaluating mares with or without endometritis. Therefore, our objective was to evaluate the blood flow of the uterine segments by Powerflow mode Doppler US, relating it to the days of the estrous cycle of mares with endometritis. The mares were separated into three groups: CG—control group with mares without endometritis; GES—group of mares with subclinical endometritis; and GEC—group of mares with clinical endometritis. With ovulation induction, the mares were monitored by transrectal B-mode US and Powerflow mode Doppler US of the uterine segments. The examinations were performed daily until the next ovulation. The subjective evaluation, performed by Powerflow mode Doppler ultrasound, proved to be efficient in detecting mares with clinical endometritis examined on days 1, 9, and 10 of the estrous cycle (*p* < 0.0001). It was found that subjective and objective evaluation of pixel quantity and intensity showed a significant, positive, and moderate correlation with ρ = 0.51 and ρ = 0.51, respectively (*p* < 0.0001). Thus, it is concluded that Powerflow Doppler ultrasound can be used to detect mares with clinical endometritis.

## 1. Introduction

Endometritis is the main equine reproductive pathology causing infertility or subfertility, which may be related to acute or degenerative inflammatory changes in the endometrium [1,2]. In fertile mares, this physiological inflammation resolves with the removal of uterine fluid, semen, and bacteria within 48 h of insemination. If this inflammation persists for more than 48 h, the pathological condition of persistent post-breeding endometritis arises. If this post-breeding endometritis does not resolve, this pathological condition can progress to acute infectious endometritis and then to chronic endometritis with endometrial degeneration [3].

The ability to maintain a uterine environment compatible with embryonic and fetal life is essential for reproductive efficiency in equine. However, the uterine environment is easily disrupted by an acute or chronic inflammatory process following aerobic and anaerobic bacterial and/or fungal invasion, which can occur during natural mating, artificial insemination, pre-mating season examinations, infection before and after parturition, and especially during a postpartum uterine cleansing period [4]. Some authors also believe that endometritis may be caused by dysbiosis of the commensal microbiome [2,5].

Multiparous and elderly mares (>15 years of age) are three times more likely to have vascular lesions such as elastosis, fibroelastosis, perivascular fibrosis, and vessel calcification when compared to nulliparous mares [6]. In addition, endometritis caused by biofilm-forming microorganisms such as Pseudomonas aeruginosa, Staphylococcus epidermis, Escherichia coli, Enterobacter cloacae, and some yeasts and fungi can trigger chronic endometritis in these mares due to a number of aggregate factors [7].

The persistence of uterine fluid accumulation for 3–5 days post-insemination, mainly due to failure of myoelectric contractility, can trigger clinical endometritis. The signs observed in clinical endometritis are echogenic fluid in the uterus and fluid in the uterus during estrus, exacerbated uterine edema, cervical orifice secretion with hyperemia, vaginitis, vaginal discharge, short inter-estrus intervals, positive or negative uterine cytology, positive uterine microbiological culture, decreased tissue perfusion resulting in an inadequate amount of neurotransmitters, hormones, and inflammatory cells to the endometrial tissue, failure of vulvar coaptation with extension of the vulva due to increased age and/or parity resulting in pneumovagina [3,6,8,9].

In subclinical endometritis, many of these signs are not observed, which can lead to misdiagnosis. Subclinical endometritis is characteristic of multiparous and adult mares. Factors such as anatomical abnormalities and uterine degeneration contribute to the presence of infection. Characteristics such as mucociliary dysfunction, scarring, cysts, and atrophy of the endometrial folds and decreased tissue perfusion are associated with subfertility in these mares [6].

The endometritis diagnosis is based on the mare’s history, physical examination, reproductive examination, and laboratory diagnosis. These tests include transrectal ultrasound, vaginal and cervical examination, endometrial cytology, microbiological culture, uterine biopsy, and in some cases, hysteroscopy. Endometrial biopsy is considered the gold standard for detecting endometritis, as it evaluates factors such as deep infiltrates and fibrosis around the glands, gland dilation, and glandular density [10]. This method can detect elastosis associated with increased calving; lymphangiectasia is secondary to vascular degeneration in chronic inflammation or in mares with impaired cervical drainage; epithelial loss, excessive exudate, and epithelial hyperplasia are indicators of chronic uterine inflammation. Degenerative changes (glandular fibrosis, glandular ectasia, and lymphangiectasia) are considered normal processes in aging (>10 years of age) [6,11]. Because endometritis is a local infection and rarely presents with systemic complications, the leukogram and inflammatory markers (fibrinogen, serum amyloid A protein, haptoglobin) are not altered [12].

With the advent of ultrasound, it became possible to detect early stages of uterine infections with abnormal patterns of edema and hyperechoic lines characteristic of air or exudate; compromised uterine clearance; and the amount and appearance of fluid present in the uterus [1,13]. The presence of 2 to 3 cm of intrauterine fluid during estrus or between 6- and 36 h post-breeding are good indicators of a mare susceptible to endometritis [3,7,14,15]. The Doppler ultrasound brought an innovative tool to the market, making it easier to ensure the diagnosis of subclinical endometritis through an assessment of local blood flow and a more accurate analysis of the physiology of the ovaries and uterus [16,17].

Based on this discourse, our group believes that there is a difference in uterine vascularization between mares with and without endometritis that can be detected by evaluating the vascularization of the uterine segments (uterine body and horns).

Therefore, our objective was to evaluate the blood flow of the uterine segments using the Powerflow mode Doppler ultrasound technique with subjective and objective evaluation of vascularization, relating it to the days of the estrous cycle of mares with endometritis.

## 2. Materials and Methods

The project was approved by the Ethics Committee for the Use of Animals of the Institute of Zootechnics of the Federal Rural University of Rio de Janeiro (CEUA-IZ/UFRRJ) under protocol number 0121-07-2021.

### 2.1. Experimental Location and Duration

This study was carried out in the equine sector (22°46′48.4″ S 43°41′03.0″ W) of the Institute of Zootechnics of the Federal Rural University of Rio de Janeiro (UFRRJ), located in the municipality of Seropédica/RJ, during the 2021/2022 and 2022/2023 breeding seasons.

### 2.2. Animals and Management

Initially, eighty mares were evaluated, from which 45 Mangalarga Marchador mares were selected, multiparous, cyclic, with a body score of 4–6 according to Henneke et al. [18] and aged between 5 and 20 years. The selected mares were separated into three experimental groups, according to the distribution criteria for the experimental groups mentioned below: CG—control group with mares without endometritis (16 animals); GES—group of mares with subclinical endometritis (15 animals); and GEC—group of mares with clinical endometritis (14 animals).

The animals used were raised on pasture, fed with forage at a consumption rate of 2% of live weight and 2 kg/day of balanced feed, and had free access to water and mineral salt. In addition, the entire health management protocol (vaccination, deworming and tick treatment) was kept up to date.

### 2.3. Criteria for Distributing the Mares into Experimental Groups

**Figure 1:**
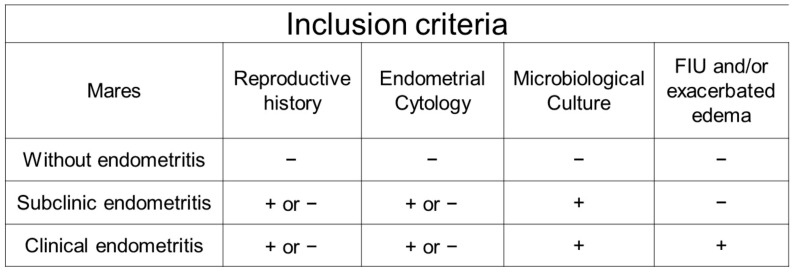
Illustrative diagram of the criteria used to distribute mares into experimental groups. FIU: intrauterine fluid.

The following criteria were used to assess and classify the mares into the experimental groups: uterine cytology examination, uterine microbiological culture examination and the presence or absence of intrauterine fluid (FIU) and/or the presence of exacerbated edema assessed by B-mode US. When uterine fluid with distension ≥ 2 cm was detected, it was considered suggestive of endometritis [6,19]. For the animals to be classified as having subclinical or clinical endometritis, a positive bacterial and/or fungal culture test was required (Figure 1).

This study did not require a positive endometrial cytology examination, as the literature suggests cytological examination has low sensitivity in detecting endometritis caused by Gram-negative bacteria. In other words, mares with bacterial endometritis caused by Escherichia coli and Pseudomonas aeruginosa tend to have a negative cytological exam, while endometritis caused by β-hemolytic Streptococcus, Staphylococcus aureus and Klebsiella pneumoniae tend to have a positive cytological exam [2,7,20].

### 2.4. Experiment

The first stage of the experiment consisted of an assess of the mares (reproductive history and ultrasound examination). The estrous cycle was then monitored until a pre-ovulatory follicle was observed (diameter ≥ 35 mm and edema ≥ 3) [19]. Based on this information, uterine samples were collected for fungal and bacterial culture and endometrial cytology, and the mares were then classified into study groups. In the mare that had a preovulatory follicle, ovulation induction was then performed. Ovulation induction was carried out using Gonadotrophin Releasing Hormone (GnRH) analogs such as deslorelin at a dose of 750 µg/mL, intramuscularly (Sincrorrelin^®^, Cravinhos, SP, Brazil) or histrelin at a dose of 250 µg/mL, intramuscularly (Strelin^®^, Botucatu, SP, Brazil) or hCG at a dose of 1000 IU, intravenously (Chorulon^®^ 5000 IU, Rahway, NJ, USA).

After ovulation detection (D0), the mares were monitored by transrectal B-mode ultrasound and Powerflow mode Doppler ultrasound of the uterine segments (left uterine horn, uterine body, and right uterine horn). These ultrasound examinations were performed every day until ovulation in the next estrous cycle, that is, a complete estrous cycle.

Five days after the second ovulation, an endometrial biopsy was carried out and a drug based on dinoprost tromethamine from the prostaglandin family was used at a dose of 5 mg/mL (volume of 1 mL), intramuscularly (Lutalyse^®^, SP, Zoetis, Brazil) for luteolysis to occur and the mare to return to heat.

#### 2.4.1. Complementary Tests

After selecting the animals, the mares were previously examined for uterine characterization and classification according to the experimental group to which they belonged. To collect the tests, the mares were contained in stock so that the vulvar and perianal region could be cleaned. Sanitization was carried out by washing the vulvar region followed by the perianal region with iodopovidone degermant, rinsing, spraying 70% alcohol and drying the vulvar region followed by the perianal region with a paper towel.

##### Fungal Culture

The first material collected was used for fungal culture. Collection was performed using double-protected stainless steel gynecological forceps for equine, previously flamed in a tray with 70% alcohol for about 5 min and cooled with sterile saline solution.

After passing through the vulva, the cervical was located for transposition of the cervix, exposing the swab to the lumen of the uterine body. After exposure, ten rotating movements were performed to collect the uterine material. The rod was then retracted into the cannula, protecting the swab, and the forceps were removed from the uterus. The swab was removed from the gynecological forceps and placed in a tube containing Stuart culture medium identified with the animal’s name and date of collection. It was then transported under refrigeration to the Microorganism Investigation Center (CIM) of the Biomedical Institute of UFF for mycological evaluation.

In the laboratory, the swabs with the samples were directly seeded into two tubes: one containing 2% Sabouraud dextrose agar (BD, Franklin Lakes, NJ, USA) and the other containing Mycosel^®^ (BD, New Jersey, USA). These tubes were incubated at room temperature (25 °C) for up to four weeks, with weekly checks for the development of colony-forming units.

If growth was observed, each colony was seeded on a Sabouraud dextrose plate to obtain pure cultures. After the growth period (approximately five to seven days), Petri dishes containing only a single type of filamentous fungus colony were separated, and macromorphological characteristics were noted: color, texture, edges, and relief.

In order to evaluate the micromorphology of the isolated filamentous fungi and identify them by genus, the microculture technique was performed. In this technique, a sterile Petri dish containing gauze and a U-shaped tube as a support for the glass slide was prepared. Each fungal isolate was then cultured on the edges of the dextrose agar block in four places with the aid of an inoculation loop, sterilized in a Bunsen burner.

The plate was covered and incubated at room temperature for a period of 5 to 7 days. When fungal growth was visually sufficient, the coverslip was removed with tweezers, then placed on a drop of lactophenol blue dye on the surface of another slide and taken to the microscope for analysis. Using lactophenol blue cotton dye, it was possible to analyze the structures of the hyphae, verifying the presence or absence of septa, the microscopic structures of the conidiophores, as well as the microscopic structures of the phialides, verifying their arrangement, which allows the differentiation of filamentous fungi into different genera.

##### Bacterial Culture

The second test was a bacterial culture. For each new test, the gynecological forceps were flamed and cooled with sterile saline solution. The same collection procedure mentioned above was used to collect this material. The swab was removed from the gynecological forceps and placed in a tube containing Stuart culture medium identified with the animal’s name and date of collection. It was then transported under refrigeration to the Gram-Positive Cocci Bacteriology Laboratory (UFF) for bacteriological evaluation.

At the UFF laboratory, the samples underwent Gram staining, morphology, and catalase testing, and were frozen for subsequent identification of bacterial species by MALDI-TOF. The day before MALDI-TOF was performed, the samples were thawed, seeded in ATS (Tryptic Soy Agar) medium, and incubated in an oven at 37 °C.

Bacterial species identification was performed by MALDI-TOF MS (Matrix-Assisted Laser Desorption/ionization Time-Of-Flight Mass Spectrometry) at the Medical Microbiology Research Laboratory at the Health Sciences Center of the Federal University of Rio de Janeiro (UFRJ).

At this stage, each isolated colony was deposited in the wells of the sample target, a metal plate supplied by the equipment manufacturer (Bruker Daltonics, Billerica, MA, USA), with the aid of sterile wooden sticks. Next, 1 μL of 70% (*v*/*v*) formic acid was added to each well of the plate and, after drying at room temperature, 1 μL of the matrix solution composed of α-cyano-4-hydroxycinnamic acid (Sigma Aldrich, St. Louis, MO, USA) was added to each well. Using the spectra obtained by the MALDI-TOF Microflex LT detector, a score was generated by the Biotyper 3.1 software for each sample analyzed.

The score generated by the Biotyper 3.1 software is a numerical value based on the comparison of the mass spectrum of an unknown sample with a database of reference spectra. The result is represented by the degree of similarity between the samples and the database. Values ≥ 2.0 are acceptable for identification at the species level; values ≥1.7 are acceptable for identification at the genus level; and <1.7 indicate no identifications or are unreliable, according to the software manufacturer’s guidelines.

In this experiment, bacterial species were identified based on the score generated by the system. Only identifications with a score ≥ 2.3 for the bacterial genus and species were considered reliable, and samples with scores between 1.9 and 2.299 indicated identification only to the genus.

##### Endometrial Cytology

The third test was endometrial cytology. In this case, a cytology brush was used to collect uterine material. For each new test, the gynecological forceps were flamed and cooled with sterile saline solution. The same procedure described in the previous tests was used to collect this material. After removing the cytology brush from the gynecological forceps, the cellular material was distributed on three microscope slides, previously identified with the animal’s name and date of collection, by imprint.

The slides were then stained with the Quick Panopticon^®^ kit (Diff Quick method), which consists of three steps: Step 1 (1 min)—performed with a fixing solvent (alcohol), responsible for dehydrating the cells present in the smear, removing water and fixing the cell structures on the slide; 2nd step (10 s)—performed with eosin dye, an acid dye that binds to the acid components present in the cytoplasm of cells, giving a pink to orange color to the plasma structures; and 3rd step (5 s)—performed with methylene blue dye, a basic dye that binds to basic components of the cell nucleus. Finally, the slides were washed in a thin stream of running water to remove excess dye and placed at an angle to dry.

The slides were read following the classification methodology described by Riddle, LeBlanc, Stromberg [21], classifying the exam as negative when the slides presented ˂2 neutrophils per 10 fields per slide; moderate endometritis when the slides presented 2–5 neutrophils per 10 fields per slide; and severe endometritis when the slides presented ≥5 neutrophils per 10 fields per slide. At the end of the reading, an average of the number of polymorphonuclear cells found in the three slides of each mare was calculated to categorize them.

##### Uterine Biopsy

Finally, an endometrial biopsy was performed on the fifth day after completion of the ultrasound examinations to avoid possible interference with vascular hemodynamics. The mares were pre-sedated with detomidine hydrochloride at a dose of 0.002 mL/kg, administered intravenously. This examination is important for evaluating the uterine layers and their vascularization associated with possible changes when compared with the Doppler ultrasound examination.

Biopsy samples were collected using Yeoman forceps, previously flamed in a tray with 70% alcohol for about 5 min and cooled with sterile saline solution. The endometrial tissue fragment, measuring around 2 cm, was removed at the bifurcation between the body and the left or right uterine horns. The samples collected were immediately placed in Bouin’s fixative for 24 h and then transferred to 10% formalin. The endometrial tissue was stained with H/E and evaluated using the Kenney and Doig scale [22], classifying samples without significant changes with endometrial tissue considered normal as category I; samples with mild scattered inflammation, mild lymphatic lacunae, and partial endometrial atrophy in the reproductive season as category IIA; samples with moderate scattered inflammation and moderate lymphatic lacunae as category IIB; and samples with severe irreversible changes with fibrosis and inflammation and marked lymphatic lacunae with deep endometrial atrophy in the reproductive season as category III.

#### 2.4.2. Ultrasound Evaluation

The mares were examined using B-mode ultrasound and Doppler ultrasound with Mindray Medical International Limited Z5 VET equipment with a 6LE5Vs Linear Rectal transducer (5.0/6.5/8.0 MHz) and performed by the same operator throughout the study.

The mares were examined by transrectal B-mode ultrasound for a scan of the general reproductive region, followed by evaluation with Powerflow mode Doppler ultrasound to observe blood perfusion of the uterine segments. The following settings were applied: gain 100, frequency 5 MHz, depth 7.4, power 100%.

**Figure 2:**
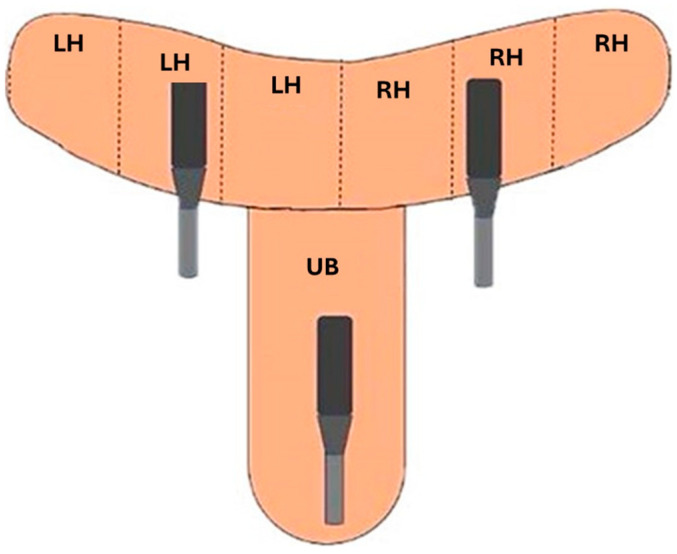
Representation of the precursor used for uterine vascular perfusion scanning. A longitudinal section of the uterine body (UB) and a cross-section of the three portions of the left uterine horn (LH) and the right uterine horn (RH) were taken. Source: personal archive.

Uterine vascular perfusion was performed by scanning the entire uterine length. Longitudinal videos of the uterine body (1 scan) and transverse videos of the left and right uterine horns (1 scan per side) were performed (Figure 2).

**Figure 3:**
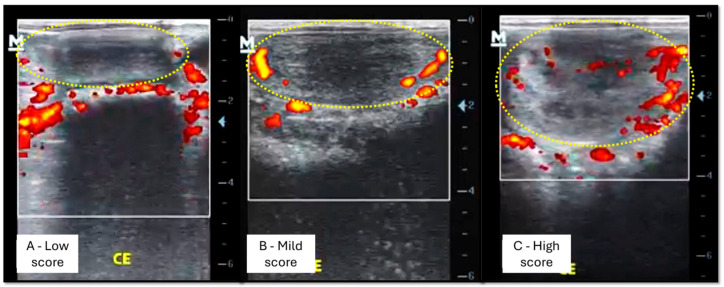
Score classification of the subjective evaluation performed simultaneously with the Powerflow mode Doppler ultrasound examination of the mares’ uterus. The yellow dotted circle delimits the uterine area where uterine vascularization was analyzed. (**A**) represents mares evaluated with a vascularized uterine area of less than 25% (low score). (**B**) represents mares evaluated with a vascularized uterine area between 25 and 49% (mild score). (**C**) represents mares evaluated with a vascularized uterine area between 50 and 75% (high score). Source: personal archive.

The subjective evaluation, the percentage (0 to 100%) of endometrial tissue with colored signs visualized during estrus and diestrus was estimated in real time. Thus, mares evaluated with a vascularized uterine area of less than 25% received a low score; from 25 to 49%, a mild score; from 50 to 75%, a high score; and above 75%, a very high score (Figure 3).

**Figure 4:**
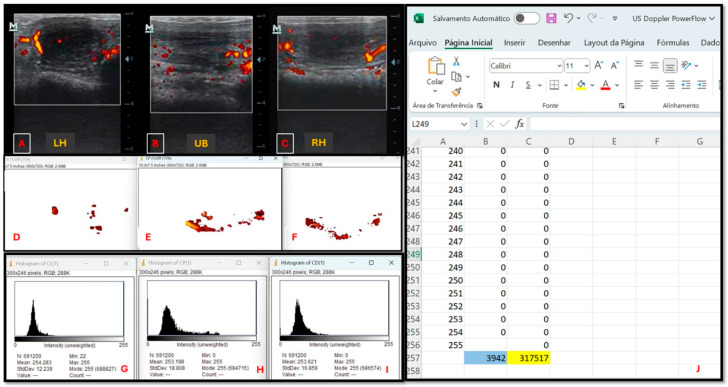
Diagram of the step-by-step process for objectively evaluating the saved video of a Powerflow mode Doppler ultrasound examination of mares’ uterus. (**A**–**C**) Images classified as having the highest uterine vascularization in the video viewed (LH: left uterine horn; UB: uterine body; RH: right uterine horn). (**D**–**F**) Images edited in Photoshop to display only uterine vascularization. (**G**–**I**) Results of the evaluation performed on each edited image, visualized by the histogram. (**J**) Histogram data in spreadsheet format, with column A representing the color palette numbering from 0 to 255 (black to white); column B representing the number of pixels in each color, with the sum of these pixels for the analyzed image highlighted in blue; and column C representing the pixel intensity in each color, with the sum of this intensity for the analyzed image highlighted in yellow. Intensity is assessed by multiplying the color palette numbering (column A) by the number of pixels in each color (column B). Source: personal archive.

The objective evaluation, the vascularization of the uterine segments, was assessed by determining the number and intensity of colored Doppler dots (degree of brightness) in a frozen image. For this assessment, the Doppler ultrasound exams were saved and evaluated using multimedia playback software on a computer. The most representative image of each uterine segment, characterized by the largest uterine area containing the greatest number of colored signals, was selected. These images were extracted and saved in TIFT format using the Photopea—Online Photo Editor^®^ program. The total number of colored dots per TIFT image was calculated using ImageJ 1.52a^®^ (National Institutes of Health, Bethesda, MD). Finally, an evaluation of the quantity and intensity of the pixels was performed based on the histogram of each modified image, and the values were averaged using Excel^®^ (Figure 4).

### 2.5. Statistical Analysis

The normality of quantitative variables was determined using the Kolmogorov–Smirnov and Shapiro–Wilk tests. To compare the means of the objective evaluation variables (pixels and intensity) obtained from the mares in the different experimental situations, analysis of variance was performed, followed by Tukey’s test for comparing means. To compare the subjective evaluation scores obtained from the mares in the different experimental situations, the Kruskal-Walli’s test was used, followed by Dunn’s multiple comparison test. To study the correlation between the subjective evaluation and objective evaluation variables (pixels and intensity), Spearman’s correlation test was used. The data was presented as: mean ± standard deviation of the mean for parametric data. The level of significance considered was *p* < 0.05.

## 3. Results

In the evaluation of the data obtained by Powerflow mode Doppler ultrasound, a line of behavior of the variables can be developed throughout the days of the estrous cycle of mares to evaluate the variables in relation to the treatment groups. Thus, the behavior of the vascularization of the uterine segments can be evaluated by subjective assessment.

**Figure 5:**
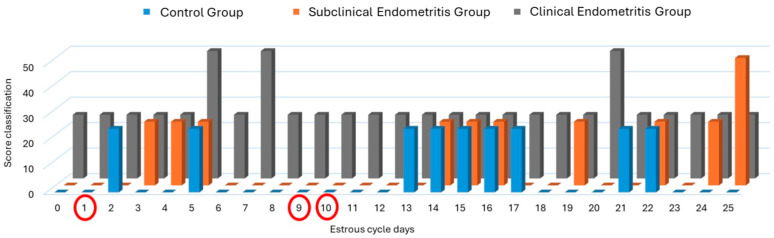
Uterine vascularization behavior through subjective assessment of uterine segments (vertical axis) during the mare’s estrous cycle (horizontal axis), divided into control group (blue line), subclinical endometritis group (orange line), and clinical endometritis group (gray line). A significant increase in uterine vascularization scores can be observed for the clinical endometritis group when compared to the subclinical endometritis group on day 01 (*p* = 0.032921); on day 09 when compared to the clinical endometritis group in relation to the subclinical endometritis and control groups (*p* < 0.05); and on day 10 when compared to the clinical endometritis group in relation to the control group (*p* = 0.033497).

That said, a significant increase in uterine vascularization scores could be observed on day 1 of the estrous cycle in the clinical endometritis group when compared to the subclinical endometritis group (*p* = 0.032921); on day 9 of the estrous cycle in the clinical endometritis group when compared to the subclinical endometritis and control groups (*p* < 0.05); and on day 10 of the estrous cycle in the clinical endometritis group when compared to the control group (*p* = 0.033497) (Figure 5).

**Figure 6:**
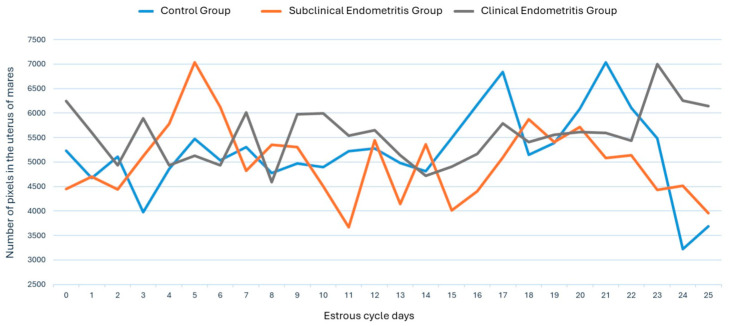
Uterine vascularization behavior through objective assessment of the pixel number of uterine segments during the mare’s estrous cycle, divided into a control group (blue line), subclinical endometritis group (orange line), and clinical endometritis group (gray line). Where no significant differences were observed between the days of the estrous cycle, the treatment groups, and the variable for objective assessment of the pixel number of uterine segments (*p* > 0.05). The control group had a mean standard deviation of 5203 ± 837.75; the subclinical endometritis group had 4995 ± 760.72; and the clinical endometritis group had 5544 ± 562.91.

**Figure 7:**
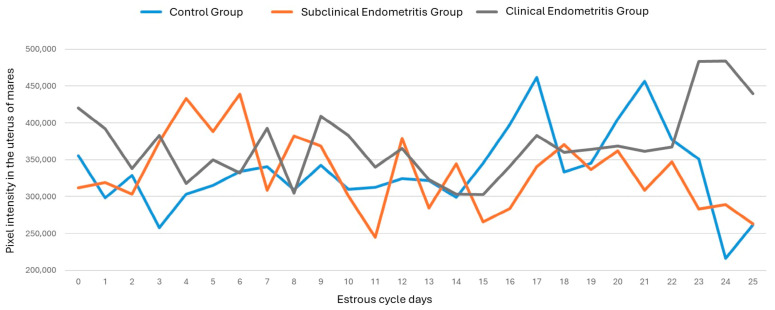
Uterine vascularization behavior through objective assessment of the pixel intensity of uterine segments during the mare’s estrous cycle, divided into a control group (blue line), subclinical endometritis group (orange line), and clinical endometritis group (gray line). Where no significant differences were observed between the days of the estrous cycle, the treatment groups, and the variable for objective assessment of the pixel intensity of uterine segments (*p* > 0.05). The control group had a mean standard deviation of 334,820 ± 54,450; the subclinical endometritis group had 332,084 ± 50,859; and the clinical endometritis group had 369,699 ± 48,625.

Similarly, the vascularization behavior of the uterine segments can be objectively assessed by evaluating the number of pixels and pixel intensity during the mare’s estrous cycle (Figure 6 and Figure 7, respectively). However, no significant differences were observed between the days of the estrous cycle, the treatment groups, and the objective evaluation variables (number of pixels and pixel intensity) (*p* > 0.05).

The subjective and objective assessments of pixel quantity showed a significant, positive, and moderate correlation with ρ = 0.51 (*p* < 0.0001), while the subjective and objective assessments of pixel intensity showed a significant, positive, and moderate correlation with ρ = 0.50 (*p* < 0.0001). In addition, the objective assessment of pixel quantity and pixel intensity showed a significant, positive, and strong correlation with ρ = 0.94 (*p* < 0.0001).

Thus, it can be observed that the uterine vascularization scores found in the subjective assessment were higher in the clinical endometritis group than those found in the control and subclinical endometritis groups (*p* ≤ 0.05). No significant difference was observed between the treatment groups in either the objective assessment of pixel quantity or pixel intensity (*p* > 0.05) (Table 1).

When analyzing the microbiological culture test, it was observed that the group with Gram-positive infection had the highest mean when compared to the group with fungal, mixed, and no infection, and was not different from the group with Gram-negative infection (*p* < 0.0001). Furthermore, in the objective evaluation for pixel quantity, a significant difference was observed between Gram-positive and Gram-negative bacterial infections and fungal and mixed infections (*p* < 0.0001). In the objective evaluation for pixel intensity, a difference can be observed between Gram-negative and mixed infections and fungal infection (*p* < 0.05) (Table 1).

In the endometrial cytology exam, a significant difference was observed between mares with negative and positive cytology exams (moderate and severe), regardless of the degree of inflammation (*p* < 0.0001). When analyzing the objective evaluation by pixel quantity and pixel intensity, a significant difference was observed associated with moderate inflammation when compared to the other groups (negative and severe) (*p* < 0.0001) (Table 1).

In the endometrial biopsy examination, both by subjective evaluation (*p* < 0.001) and objective evaluation (*p* < 0.0001), a significant difference is observed in classification IIA in relation to classifications I and IIB (Table 1).

## 4. Discussion

In the evaluation by US Doppler Powerflow mode, with subjective assessment of the vascularization of the uterine segments, the difference found between mares with clinical endometritis in relation to mares with subclinical endometritis or without endometritis can be highlighted. This variation can be detected on day 1 for mares with subclinical endometritis, on day 9 for mares with subclinical endometritis and/or without endometritis, and on day 10 for mares without endometritis. These findings can be linked to the peak concentration of P4 that occurs between days 8–10 of the mare’s estrous cycle [23] together with the vasoconstriction action that P4 performs in the uterine region during the diestrus [4].

However, when mares with clinical endometritis are mentioned, it is noted that the action of defense cells such as neutrophils, prostaglandins, oxytocin, and pro- and anti-inflammatory cytokines occur at the beginning of the diestrus to provide a uterine environment suitable for possible pregnancy, but this action fails. There is an increase in anti-inflammatory actions, causing the endometrium to release prostaglandins rapidly, leading to early luteolysis [3,24,25]. This explains the increased vascularization of the uterine segments of mares with clinical endometritis, as they return to estrus earlier than other classes.

Based on the results found in this study, we believe that subjective assessment is more appropriate for assessing the degree of endometritis: acute or chronic, requiring a greater or lesser blood supply, respectively, than for classifying it as clinical or subclinical [2,12].

Combining this information, we observed that in the endometrial cytology exam, it was possible, through subjective evaluation of Powerflow mode Doppler ultrasound, to distinguish mares with and without endometritis based on negative and positive cytology classifications (moderate and severe), respectively. Based on the microbiological culture test, it is also assumed that subjective evaluation is indicated in the identification of acute endometritis, given the difference observed by Gram-positive bacteria, which have a higher classification of uterine vascularization consistent with a greater blood supply for the transport of defense cells to fight uterine infection.

Thus, it is possible to correlate endometrial cytology and microbiological culture information with acute endometritis generated by Gram-positive bacteria, which is consistent with what has already been described in the literature, where Gram-positive bacteria show a correlation of positive endometrial cytology with a high number of neutrophils and inflammatory exudate production [6,7].

Regarding the tabulated data from the objective analysis of the vascularization of the uterine segments to assess the quantity and intensity of the pixels in the Doppler images, no significant difference was observed between the treatment groups, and it is not indicated as a complementary diagnostic method for identifying mares with and without endometritis.

However, this method was important for identifying mares with moderate endometrial cytology, endometrial biopsy classification IIA, and was a priority for mares with Gram-positive bacterial infection (higher number of pixels) and mares with fungal infection (higher pixel intensity). As reported in the literature, the speed and quantity of blood flow are related to the pixel intensity (lighter colors) observed in ultrasound images [26], i.e., they are related to acute processes.

Among the variables studied and analyzed, it was noted that the subjective and objective assessments of both quantity and intensity showed a significant, positive, and moderate correlation. In view of this, we can consider the subjective assessment performed by a trained operator as a method indicated as a complementary diagnostic method that corroborates specific days of the estrous cycle to aid in the patient’s final prognosis.

## 5. Conclusions

The subjective evaluation, performed by Powerflow mode Doppler ultrasound, proved to be efficient in detecting mares with clinical endometritis examined on the 1st, 9th, and 10th days of the estrous cycle. Also, based on the microbiological culture test, subjective evaluation can be recommended as a complementary diagnostic method in the identification of acute endometritis.

However, the objective evaluation of the vascularization of the uterine segments to assess the quantity and intensity of the pixels in the Doppler images did not show any significant differences and is not recommended as a complementary diagnostic method for mares with and without endometritis. However, it can be recommended as a complementary diagnostic method in the identification of acute endometritis associated with biofilm-producing microorganisms.

Due to the reported correlation between subjective and objective evaluation of both quantity and intensity, we can consider subjective evaluation performed by a trained operator to be a method indicated as a complementary diagnosis to aid in the final prognosis of the patient related to the presence or absence of endometritis.

We also thank FAPERJ for the scholarship that enabled us to continue our research, and the companies Reproduxx^®^ and Botupharma^®^ for supplying part of the material needed for our research.

## Figures and Tables

**Table 1:** Uterine vascularization subjective evaluation and quantity and intensity of pixels objective evaluation compared to the treatment groups, microbiological culture examination, endometrial cytology examination, and endometrial biopsy of this study.

	n	Subjective Assessment	Number of Pixels	Intensity of Pixels
**Treatment Groups**				
**GC**	16	Low perfusion ^a^	5212 ± 2452 ^a^	33,187 ± 170,984 ^a^
**GES**	15	Low perfusion ^a^	5069 ± 2534 ^a^	436,207 ± 15,772,230 ^a^
**GEC**	14	Mild perfusion ^b^	5475 ± 2856 ^a^	362,201 ± 208,170 ^a^
**Microbiological culture**				
**0**	16	Low perfusion ^a^	5221 ± 2456 ^ac^	333,600 ± 171,370 ^ab^
**F**	4	Low perfusion ^ac^	4187 ± 1441 ^bd^	276,727 ± 109,125 ^b^
**G+**	6	Mild perfusion ^b^	5480 ± 2101 ^ac^	357,362 ± 155,629 ^ab^
**G−**	9	Mild perfusion ^bc^	5837 ± 3207 ^c^	379,045 ± 235,528 ^a^
**M**	10	Low perfusion ^a^	5015 ± 2763 ^ad^	373,168 ± 452,380 ^a^
**Endometrial cytology**				
**Negative**	33	Low perfusion ^a^	5048 ± 2257 ^a^	331,502 ± 187,156 ^a^
**Moderate**	10	Mild perfusion ^b^	6013 ± 3565 ^b^	429,380 ± 452,930 ^b^
**Severe**	2	Mild perfusion ^b^	4673 ± 1633 ^a^	297,283 ± 122,428 ^a^
**Endometrial biopsy**				
**I**	2	Low perfusion ^a^	4326 ± 1237 ^a^	288,050 ± 103,864 ^a^
**IIA**	13	Mild perfusion ^b^	6013 ± 3420 ^b^	415,687 ± 413,469 ^b^
**IIB**	18	Low perfusion ^a^	4885 ± 2078 ^a^	330,696 ± 198,668 ^a^
**III**	12	Low perfusion ^ab^	4885 ± 2079 ^a^	325,753 ± 159,811 ^a^

Different letters between lines means statistical difference (*p* ≤ 0.05). GC: control group; GES: subclinical endometritis group; GEC: clinical endometritis group; 0: animals without infection; F: animals with fungal infection; G+: animals with Gram-positive bacterial infection; G−: animals with Gram-negative bacteria infection; M: animals with mixed infection.

## Data Availability

The data that support the findings of this study are available from the corresponding author upon reasonable request.
